# From the Environment to the Host: Re-Wiring of the Transcriptome of *Pseudomonas aeruginosa* from 22°C to 37°C

**DOI:** 10.1371/journal.pone.0089941

**Published:** 2014-02-24

**Authors:** Mariette Barbier, F. Heath Damron, Piotr Bielecki, María Suárez-Diez, Jacek Puchałka, Sebastian Albertí, Vitor Martins dos Santos, Joanna B. Goldberg

**Affiliations:** 1 Department of Microbiology, Immunology, and Cancer Biology, University of Virginia, Charlottesville, Virginia, United States of America; 2 Synthetic and Systems Biology Research Group, Helmholtz Centre for Infection Research, Braunschweig, Germany; 3 IUNICS, University of the Balearic Islands, Palma de Mallorca, Spain; 4 Systems and Synthetic Biology, Wageningen University, Wageningen, Netherlands; 5 LifeGlimmer GmbH, Berlin, Germany; 6 Department of Pediatrics, and Center for Cystic Fibrosis Research, Emory University School of Medicine, Children’s Healthcare of Atlanta, Inc., Atlanta, Georgia, United States of America; East Carolina University School of Medicine, United States of America

## Abstract

*Pseudomonas aeruginosa* is a highly versatile opportunistic pathogen capable of colonizing multiple ecological niches. This bacterium is responsible for a wide range of both acute and chronic infections in a variety of hosts. The success of this microorganism relies on its ability to adapt to environmental changes and re-program its regulatory and metabolic networks. The study of *P. aeruginosa* adaptation to temperature is crucial to understanding the pathogenesis upon infection of its mammalian host. We examined the effects of growth temperature on the transcriptome of the *P. aeruginosa* PAO1. Microarray analysis of PAO1 grown in Lysogeny broth at mid-exponential phase at 22°C and 37°C revealed that temperature changes are responsible for the differential transcriptional regulation of 6.4% of the genome. Major alterations were observed in bacterial metabolism, replication, and nutrient acquisition. Quorum-sensing and exoproteins secreted by type I, II, and III secretion systems, involved in the adaptation of *P. aeruginosa* to the mammalian host during infection, were up-regulated at 37°C compared to 22°C. Genes encoding arginine degradation enzymes were highly up-regulated at 22°C, together with the genes involved in the synthesis of pyoverdine. However, genes involved in pyochelin biosynthesis were up-regulated at 37°C. We observed that the changes in expression of *P. aeruginosa* siderophores correlated to an overall increase in Fe^2+^ extracellular concentration at 37°C and a peak in Fe^3+^ extracellular concentration at 22°C. This suggests a distinct change in iron acquisition strategies when the bacterium switches from the external environment to the host. Our work identifies global changes in bacterial metabolism and nutrient acquisition induced by growth at different temperatures. Overall, this study identifies factors that are regulated in genome-wide adaptation processes and discusses how this life-threatening pathogen responds to temperature.

## Introduction

*Pseudomonas aeruginosa* is a ubiquitous microorganism infecting a wide range of hosts, including plants, insects, and mammals. This bacterium is also a major human nosocomial pathogen, responsible for acute or chronic infections in patients with burn wounds or with significant underlying diseases such as cystic fibrosis, respectively. The success of *P. aeruginosa* as a pathogen relies on its highly versatile genome, adaptive capability, and expression of a large array of virulence factors [1], [2]. The bacterium senses changes in its environment, such as nutrient concentration, pH, temperature, or osmotic pressure, and responds to them accordingly. The adaptation response involves re-programming of regulatory and metabolic networks, and activation of differential expression of the genes the products of which are necessary to support successful colonization and growth. The expression of unnecessary genes is also reduced to decrease their metabolic cost [3].

*P. aeruginosa* is a mesophilic bacterium, growing at temperatures ranging from 4°C to over 42°C, with an optimal growth temperature of 37°C [4]. Temperature variations are frequently encountered in the environment and have been associated to profound modulation of gene expression in *P. aeruginosa* and other microorganisms. Microbial response to temperature changes includes variations in metabolism, membrane structure, motility, chemotaxis, and other general adaptive responses [5]–[8]. Several plant and human pathogens, including *P. aeruginosa,* have also been shown to express factors involved in host colonization [9], [10], including phenazines, lipopolysaccharide O-antigen, quorum-sensing, and trimethyl-lysine modification of the elongation factor EF-Tu in a temperature-dependent manner [11]–[13].

*P. aeruginosa* infections are mainly acquired through direct contact with contaminated water or other sources in the environment, inhalation of aerosols, contaminated medical devices or equipment. *P. aeruginosa* persists on these surfaces at ambient temperature, and its transition to the host is associated with increased temperature [14], [15]. Understanding the mechanisms of this adaptive response would help in explaining the persistence of this microorganism in the environment as well as identify the factors involved the successful host colonization.

We hypothesize that changes in temperature cause coordinated gene expression that support the metabolic transition of this microorganism and its adaptation to the mammalian host. In this study, we used microarray analysis and compared the *P. aeruginosa* PAO1 transcriptome in Lysogeny broth at 22°C and 37°C to enhance our understanding of *P. aeruginosa* adaptation to temperature changes. To identify thermo-regulated factors crucial for the switch of environment and host colonization, we also compared our data to two different datasets of *P. aeruginosa*: a) PA14 grown at both 28°C and 37°C [13], and b) *ex vivo* transcriptional profiles of clinical isolates from burn wound infections compared to the growth of these isolates in Lysogeny broth at 37°C [16]. Our results indicate that temperature changes are responsible for the differential regulation of a large proportion of the genome that trigger important alterations in bacterial metabolism, replication and nutrient acquisition. Our data also provide new insights into amino acid utilization and pH regulation at these temperatures.

## Methods

### Bacterial Strains and Culture Conditions

The *P. aeruginosa* type strain PAO1 was kindly provided by Dr. Antonio Oliver (Hospital Son Espases, Spain) and was used for all experiments. PAO1 was grown in 50 ml Lysogeny broth (10 g of tryptone (Difco), 5 g of yeast extract (Difco) and 5 g of NaCl (Fisher) per liter) in 250 ml flasks at 22°C and 37°C under constant shaking at 180 rpm. Unless otherwise specified, all reagents were purchased from Sigma Aldrich (St. Louis, MO).

### Total RNA Isolation and Hybridization

*P. aeruginosa* PAO1 was grown in three independent cultures, as mentioned above. Once the cultures reached OD_600_ = 3 (12 h of growth at 37°C and 15 h at 22°C), the cells were harvested and stabilized with RNA protect (Qiagen, Carlsbad CA). RNA was extracted as described previously [17] using RNeasy mini purification kit (Qiagen, Carlsbad CA) and following the manufacturer’s instructions. RNA was extracted as described previously [17] using RNeasy mini purification kit (Qiagen, Carlsbad CA) and following the manufacturer’s instructions. RNA obtained from three independent cultures were pooled and amplified using the MessageAmp II Bacteria procedure (Ambion, Austin TX). A total of 1 µg of RNA was polyadenylated and the complementary strand was synthesized by reverse transcription using T7-oligo-dT. RNase H was used to remove RNA from the solution and the second DNA strand was synthesized with DNA polymerase. cDNA was transcribed to amplified RNA (aRNA) using a T7-RNA polymerase and biotinylated dUTP and dCTP. aRNA quality, concentration, and possible degradation was measured using a 2100 BioAnalyzer (Agilent Technologies, Santa Clara CA). 5× fragmentation buffer was used to fragment the aRNA and 6.5 µg of fragmented biotinylated aRNA were used per chip. Samples were analyzed in triplicate on *P. aeruginosa* GeneChips® (Affymetrix, Santa Clara CA) following the procedure established by the manufacturer.

### Microarray Data Normalization and Analysis

The data generated were analyzed using Bioconductor microarray analysis suite (http://www.bioconductor.org) [18]. The quality of all chips was assessed by fitting a linear model to the probe level data using the fitPLM function from the affyPLM package. Expression values were computed using the Robust Multichip Average algorithm. Differentially expressed genes were identified using the Rank Products algorithm [19]. The value of 0.05 was accepted as a cut-off for the proportion of false positive.

The data obtained were classified using the PseudoCAP function class assignment and the primary cellular localization assignment of the PAO1 genome annotation published by the *Pseudomonas* Genome Database (www.pseudomonas.com) [20]. The transcriptome data were mapped to metabolic pathways using the Kyoto Encyclopedia of Genes and Genomes database (www.genome.jp/kegg) [21]. Microarray data have been deposited to the National Center for Biotechnology Information’s Gene Expression Omnibus (GEO) and can be accessed through GEO series accession number GSE51409.

### Comparison of the Changes of Gene Expression in Different Datasets

The list of genes the expression of which was differentially regulated between 28°C and 37°C in rich medium was extracted from the published study from Wurtzel *et al.* [13]. The list of genes differentially regulated in *ex vivo* samples from *P. aeruginosa* burn wound infections compared to the growth of the infection isolates in rich medium at 37°C was obtained from Bielecki *et al.* [16]. These data were analyzed using CLC MainWorkbench 6 (CLC, Aarhus, Denmark, www.clcbio.com), compiled in Microsoft Excel 2010 and overlaps between the dataset were determined using vertical search and sorting functions.

### GO Enrichment Analysis of the Different Datasets

Twelve groups of genes identified were used for comparison in a GO enrichment analysis. The groups were defined as: A22a and A37a (all the genes overexpressed at 22°C and 37°C, respectively, in this study), A22 and A37 (genes exclusively overexpressed at 22°C and 37°C, respectively, in this study), B28a and B37a (all the genes overexpressed at 28°C and 37°C, respectively, in [13]), B28 and B37 (genes exclusively overexpressed at 28°C and 37°C, respectively, in [13]), CBWa and C37a (all the genes overexpressed in burn wounds and at 37°C, respectively, in [16]), CBW and C37 (genes exclusively overexpressed in burn wound and at 37°C, respectively, in [16]). The regions of overlap between each group were also analyzed. The limited number of genes present in three sets overlaps was too small to be included in the analysis. The GO annotation for *P. aeruginosa* was obtained from the UniProt-GOA database [22]. GO enrichment analysis was performed using a hypergeometric function to model the probability density, as implemented in the GOHyperGAll function [23]. *P_hyper_*, the p-values for the enrichment and *P_adj_*, the p-values corrected for multiple testing were determined. Pathways with *P_adj_* inferior to 0.05 were considered statistically significant.

### Validation of Array Data by Reverse Transcriptase-quantitative PCR (RT-qPCR)

*P. aeruginosa* PAO1 was grown in Lysogeny broth in three independent cultures at 22°C and 37°C, as described above. Samples were taken when OD_600_ reached 3.0 and total RNA was purified and DNase-treated. DNase-treated RNA was used as a template in a 35-cycle PCR to assess the presence of contaminating DNA. cDNA was synthesized from 1 µg of total RNA using a TaqMan reverse transcription kit (Applied Biosystems, Austin TX) following the manufacturer’s instructions. qPCR were setup in 20 µl volumes using 1 µl of cDNA, 10 µl of 2× FastStart Universal SYBR green qPCR master mix (Roche, Basel, Switzerland), and 4 pmol of each primer (Table S1). qPCRs were performed in 96-well plates in an ABI Prism 7900HT Fast Real Time thermocycler with the following program: 10 min incubation at 95°C, 40 cycles of 15 s at 95°C, 1 min at 60°C and a denaturation step. Threshold cycle (CT) values were collected with a manual threshold of 0.2. Each gene targeted was analyzed in triplicate. CT were converted to a relative transcript number by the equation n = 2^(40−CT)^ and then standardized to the determined value for *omlA* as previously described [24]. These normalized values were used to determine fold changes and data were analyzed using a one sample t-test with the software GraphPad Prism 5.0.

### Iron Quantification

*P. aeruginosa* PAO1 was grown in six independent cultures, as described above. At regular intervals, samples were taken and centrifuged for 10 min at 5,000 rpm. The concentration of Fe^2+^ and Fe^3+^ was measured in culture supernatant using a method previously described [25]. Briefly, pH was measured and 2 ml of sample were treated with 200 µl of 10 mM ferrozine in 100 mM ammonium acetate and mixed thoroughly. The absorbance of each sample was measured at 562 nm. 800 µl of each sample were then mixed with 150 µl of 1.4 M of hydroxylamine hydrochloride in 2 M hydrochloric acid. Samples were mixed and incubated at ambient temperature for 10 min. Finally, 50 µl of 10 M ammonium acetate were added and absorbance was measured at 562 nm. A solution of 17.86 mM of FeCl_3_ in Lysogeny broth was used as calibration standard. Fe^2+^ and Fe^3+^ concentrations were determined using the Lambert-Beer law [25]. All measurements were performed on six individual biological replicates with two technical replicates each. Data were analyzed using a paired two-tailed t-test with the software GraphPad Prism 5.0.

### Pyoverdine Assay

Pyoverdine production was measured as previously described [13]. Briefly, *P. aeruginosa* PAO1 was grown in triplicate, as described above, and cultures were centrifuged for 10 min at 5,000 rpm. Pyoverdine absorbance was measured in culture supernatant at 400 nm. Data were analyzed using a paired two-tailed t-test with the software GraphPad Prism 5.0. Pictures of culture supernatant after 24 h of incubation were taken with a Canon EOS450D.

### Measurement of Bacterial Growth on Different Sources of Carbon and Nitrogen

*P. aeruginosa* PAO1 was grown in 10 ml Lysogeny broth overnight at 37°C. Bacterial cells were washed twice with M9 minimal medium and resuspended in 10 ml M9 minimal medium. M9 minimal media containing 300 mM of arginine, succinic acid, or glutamic acid were inoculated with 10^6^ colony-forming units per ml of PAO1. Cultures were setup in microtiter plates in volumes of 200 µl. Plates were incubated under constant agitation and absorbance measurements were taken every 15 min at 595 nm using a TECAN Infinite F500 (Männedorf, Switzerland).

## Results and Discussion

### Transcriptome Analysis

During the establishment of an infection, *P. aeruginosa* experiences a switch from ambient to body temperature (around 37°C). The bacterial factors expressed during this transition have been shown to be critical for bacterial virulence and for infection [9], [10]. Surprisingly, however, only a few studies have looked at the influence of temperature on the virulence and adaptation of *P. aeruginosa* [7], [26], [27]. Here we hypothesized that temperature changes trigger coordinated gene expression such that would facilitate the metabolic transition of the microorganism upon infection. Given that *P. aeruginosa* is mostly an environmental microorganism, we expect many virulence factors critical for infections in mammalian hosts to be up-regulated when *P. aeruginosa* is grown at body temperature. To better understand the consequences of temperature changes on *P. aeruginosa* fitness and virulence, the transcriptome of *P. aeruginosa* type strain PAO1 was studied through microarray analysis. RNA was extracted from PAO1 grown in Lysogeny broth at mid-exponential phase at 22°C and 37°C and analyzed using PAO1 Affymetrix microarrays. Overall, a total of 357 genes were found to be significantly differentially regulated, which indicates that 6.4% of the genome of PAO1 is temperature regulated under these conditions. 175 genes were found to be significantly activated and 182 were repressed at 22°C compared to 37°C (Table S2). Interestingly, another study using a different approach to compare differences of gene expression in *P. aeruginosa* strain PA14 at 28°C and 37°C reported a different set of temperature-induced genes, but an overall identical percentage of total differentially regulated genes [13].

### Temperature Change Induces Global Differential Regulation of the Transcriptome

**Figure 1:**
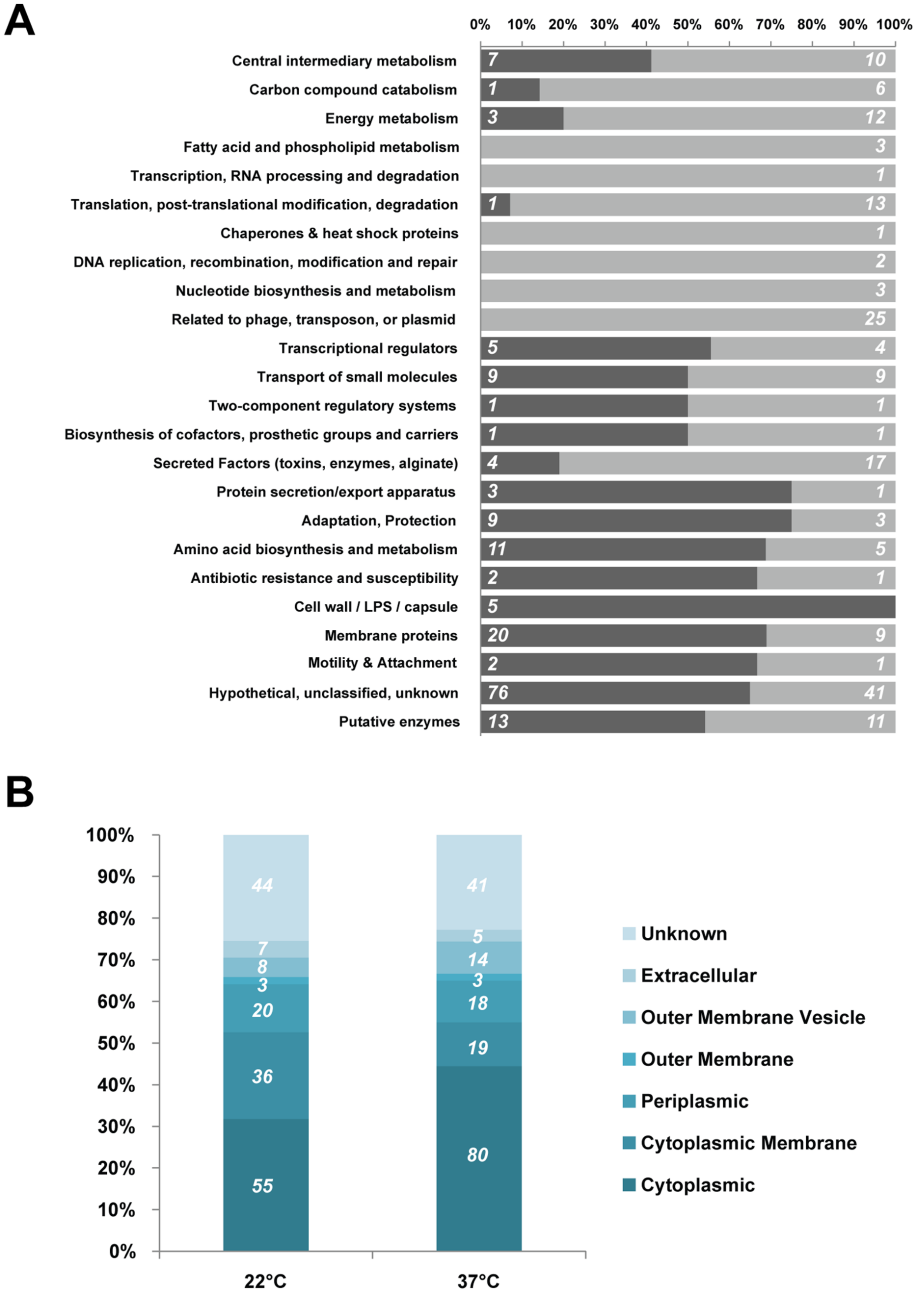
Functional analysis of differential gene expression at 22°C and 37°C. A. Classifications of the genes showing a significant differential expression in microarray analysis at 22°C or 37°C based on PseudoCAP function class assignments [28]. The percentage of genes down-regulated in PAO1 at 22°C compared to 37°C is indicated in dark grey and the percentage of the genes up-regulated is indicated in light grey. The absolute number of genes differentially regulated under these conditions in each class is indicated on the bars. B. Subcellular localization of the products of the genes differentially regulated at 22°C and 37°C. Data represent the primary localization assignment of the gene products based on PAO1 genome annotation of the *Pseudomonas* Genome Database (www.pseudomonas.com) [20]. Gene products of unknown location were removed from this analysis.

To gain insights into the implication of temperature-induced gene regulation on bacterial metabolism, the genes observed to be differentially expressed were classified based on PseudoCAP function class assignments [28] (Figure 1A and Table S2). The results indicate that genes involved in energy metabolism through carbon and fatty acid degradation are down-regulated at 22°C. Furthermore, genes which products are involved in protein translation and post-translational modification (ribosomal proteins RplL, RplT, RpmA, RpmB, RpmE, RpmG, RpmH, RpmJ, RpsL, RpsN, RpsT, the RNA helicases PA0428 and PA3344, or the heat-shock protein IbpA and DsbG), DNA replication (DNA polymerase HolA), and nucleotide biosynthesis (NrdA, NrdB, GuaA, GuaB, and PA0196) are also down-regulated at 22°C. On the other hand, genes which products are associated with adaptation (pyoverdine biosynthesis), protection (response to osmotic pressure and oxidative stress), motility (chemotaxis and aerotaxis), or amino acid metabolism (arginine and ornithine), are up-regulated at 22°C compared to 37°C. According to these results, genes involved in energy metabolism and bacterial replication are up-regulated when the organism grows at 37°C, which correlates with a higher growth rate of the bacterium at 37°C (Figure S1).

Among the genes differentially regulated (Figure 1B), the genes which encode products located in the periplasm and cell membrane of *P. aeruginosa* PAO1 are preferentially expressed at 22°C. These genes include *pctB* and PA4915 which are involved in bacterial chemotaxis [29], and are up-regulated at 22°C. These results correlate with the fact that environment sensing in sub-optimal growth conditions is crucial for the adaptation, survival and successful colonization of the niche.

The results obtained corroborate previous studies suggesting that the mammalian host temperature, 37°C, is more optimal for *P. aeruginosa* growth [4], and show that the processes involved in energy metabolism and cell replication are highly up-regulated at this temperature. However, other processes involved in cell adaptation and persistence are up-regulated at 22°C. This suggests that, even though *P. aeruginosa* cell division rate is lower at this 22°C, this temperature allows for the expression of factors essential for bacterial survival and persistence outside the mammalian host, such as in soil and water. No changes were observed in the expression of other *P. aeruginosa* heat-shock proteins such as DnaK, Lon, HptG, GroEL or RpoH [30]. These results suggest that the differences of temperatures used in this study did not elicit a heat-shock response.

### Proteases and Secreted Factors are Differentially Regulated at 22°C and 37°C

**Figure 2:**
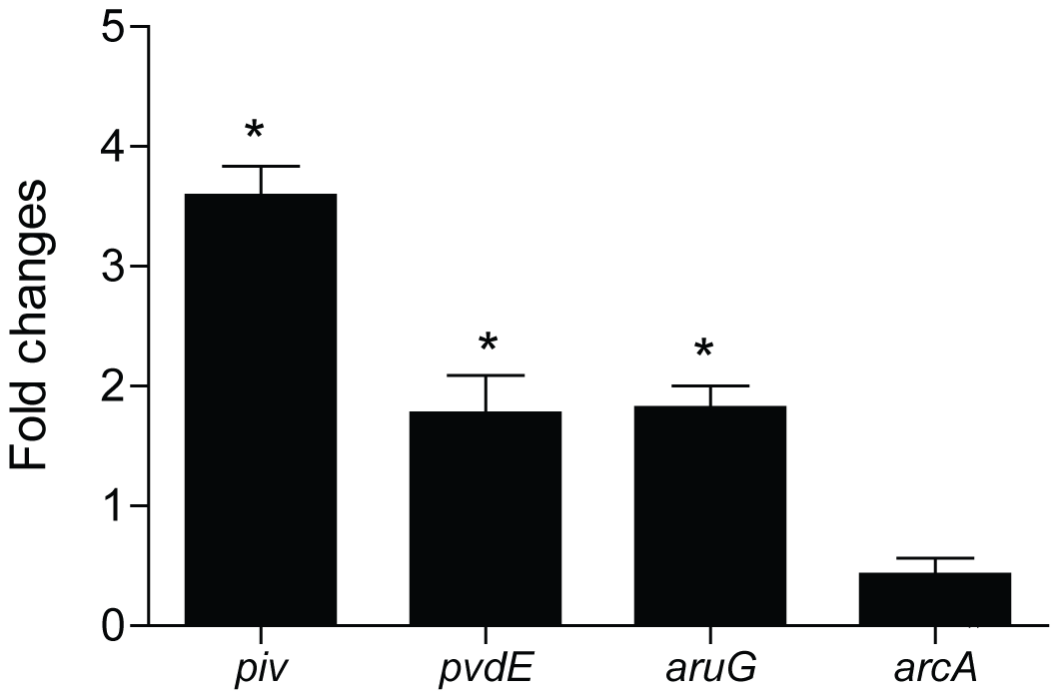
RT-qPCR analysis of temperature dysregulated genes. Analysis of the expression of control genes by RT-qPCR to validate microarray analysis. Each gene was analyzed in triplicate and data are represented as fold changes at 22°C compared to 37°C. Data were analyzed using a sample two-tailed t-test. Genes significantly dysregulated are denoted with an asterisk.

The acquisition of nutrients is often challenging for bacterial cells. Extracellular proteases play a crucial role in nutrient scavenging in *P. aeruginosa*. The gene *piv* encodes the protease IV involved in protein degradation and showed the largest differences in expression when grown at 22°C compared to 37°C (72-fold changes) (Table S2). *piv* expression at both temperatures was also analyzed by RT-qPCR. The results obtained show that *piv* is significantly up-regulated at 22°C compared to 37°C and corroborate the data obtained in the array analysis (p = 0.0045) (Figure 2). Similar results were obtained in another study, where *piv* was shown to be up-regulated at 25°C compared to 37°C [26]. Furthermore, this gene has been recently shown to be regulated by quorum-sensing, an important factor involved in bacterial adaptation [31], [32]. Its high expression at 22°C is most likely the result of the changes *lasI* expression, which shows a decrease in expression by –3.26 fold at 22°C versus 37°C. Comparison of the list of genes regulated by temperature with the quorum-sensing regulon [33] indicates that there are a total of 37 other genes the expression of which is controlled by both temperature and quorum sensing (Table S3).

Interestingly, most of the genes classified in PseudoCAP as secreted factors show a decreased expression at 22°C, including alkaline protease (*aprX, aprD, aprE, aprF*), elastase (*lasA*) and exoenzymes (*exsC, exsE, exsB, exsD,* and *exoS)* (Table S2). These genes encode well-described virulence factors in *P. aeruginosa* secreted by type I, II, and III secretion systems, respectively, of which some are quorum sensing-regulated (*aprD* and *lasI*) (Table S4). It is not surprising that *P. aeruginosa*, an environmental microorganism, does not express these virulence factors at ambient temperatures. The results obtained indicate that the expression of these genes is potentially triggered by the increase in temperature associated to the transition from the environment to the host.

### Environmental Growth Temperature Induces Global Changes in Energy and Amino Acid Metabolism

**Figure 3:**
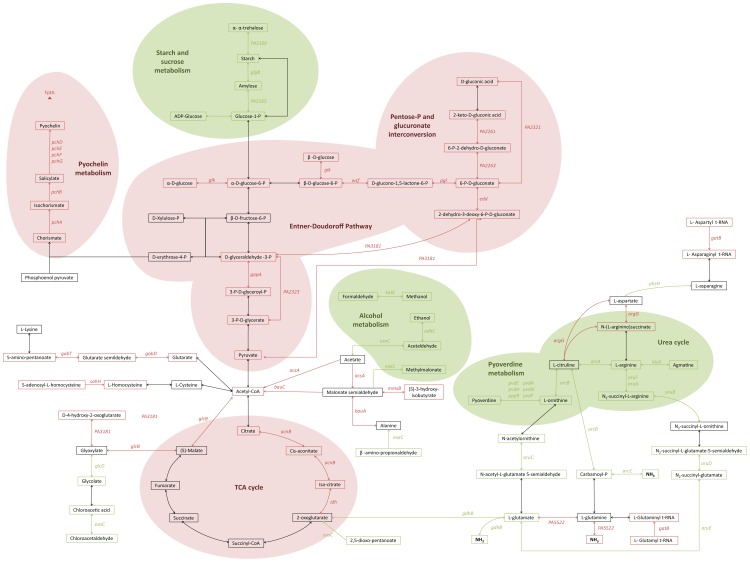
Pathways of *P. aeruginosa* differentially regulated at 22°C and 37°C. Graphic representation of the global changes in *P. aeruginosa* PAO1 metabolism detected by microarray analysis. The genes significantly dysregulated at 22°C and 37°C were mapped to metabolic pathways using the Kyoto Encyclopedia of Genes and Genomes database [21]. Genes up-regulated at 22°C (green) and genes up-regulated at 37°C (red) are italicized next to the reaction catalyzed by their products. Green and red areas represent pathways globally up-regulated at 22°C and 37°C, respectively.

PseudoCAP function analysis of the data indicates that the expression of the genes involved in bacterial metabolism and energy production substantially increased at 37°C compared to 22°C. The results obtained in the microarray analysis were mapped to metabolic pathways using the Kyoto Encyclopedia of Genes and Genomes database [21]. Overall, most of the genes involved in glucose assimilation through the Entner-Doudoroff pathway and pentose phosphate and glucuronate interconversion pathway are up-regulated at 37°C compared to 22°C. Alanine, lysine and cysteine degradation feeding the pool of acetyl-CoA, together with part of the TCA cycle were also found to be up-regulated (Figure 3). These results are probably linked to the increased growth rate and metabolic activity of the bacterium at 37°C. The pathways down-regulated at 37°C compared to 22°C include starch, sucrose and alcohol metabolic pathways. Arginine, ornithine and glutamate metabolism genes are also down-regulated at 37°C.

Arginine, ornithine and glutamate are important sources of carbon and nitrogen that can be degraded in *P. aeruginosa* through the arginine deiminase (ADI, *arcDABC*) pathway. Additionally, arginine can also be degraded through the arginine succinyltransferase (AST, *aruCFGDBE*), and arginine decarboxylase (ADC, *speA*, *aguBA*) pathways. Interestingly, both the AST and the ADI pathways were up-regulated at 22°C (Figure S2). These results were confirmed by RT-qPCR showing a significant activation of *aruG* expression at 22°C compared to 37°C (*p* = 0.0096) (Figure 2). Arginine degradation is controlled in *P. aeruginosa* by the two-component response regulator CbrA/CbrB, which induces the expression of *argR* and the small RNA CrcZ in presence of arginine [34], [35]. ArgR induces the expression of the operon *aruFGDBE* [36] (Figure S3). The *arcDABC* operon is also induced in oxygen limited environment by Anr, a transcriptional activator of protein expression in anaerobiosis [37], and in the presence of arginine through the binding of ArgR (Figure S3) [34], [38]. In this study, no changes were observed in the expression of *cbrAB* or *argR*. Furthermore, the expression of *anr* and some Anr-induced genes such as *uspKLMNO* and *hcnA* [39], were not affected by temperature changes. Only *dnr* expression, induced by Anr, is up-regulated at 22°C compared to 37°C in PAO1. Activation of *arcD* transcription has been shown to be Anr specific and is not regulated by Dnr [40]. These results indicate that none of the previously described regulators involved in the regulation of arginine degradation appear to be involved in the up-regulation of the expression of the AST and ADI pathways in *P. aeruginosa* at 22°C.

Other members of the genus *Pseudomonas* have been shown to compensate for the loss of enzymatic activity by overexpression of some of its enzymes at lower growth temperatures [41]. To determine if the differences observed in the transcriptome analysis correlate with differences of utilization of arginine at different temperatures, PAO1 was cultivated at 22°C and 37°C in minimal medium supplemented with arginine, glutamate or succinic acid (Figure S4). Results were normalized with growth at each temperature in Lysogeny broth to correct the temperature differences in growth rate observed at both temperatures. Although the expression of arginine degradation genes is increased at 22°C, the relative ability of PAO1 to grow at this temperature on arginine is unchanged (Figure S4A) compared to 37°C. However, PAO1 grew relatively faster on L-glutamate at 22°C compared to 37°C, which also correlates with the increase of expression of the enzymes involved in L-glutamate degradation at that temperature (Figure S4C). These results suggest that overexpression of the L-glutamate metabolism gene *gdhB* at 22°C compared to 37°C is important for nitrogen and carbon assimilation and facilitates bacterial growth at that temperature. The overexpression of the genes the products of which are involved in the ADI and AST pathway is not sufficient to increase bacterial growth on arginine as sole source of nitrogen and carbon at 22°C. We speculate that the overexpression of these genes at 22°C might be due to an effort to compensate for a lower enzymatic activity of these enzymes at 22°C. As control, PAO1 was also grown at 22°C and 37°C in minimal medium supplemented with succinic acid. PAO1 relative growth was faster at 37°C than at 22°C, which correlates with the activation of the expression of the enzymes involved in the TCA cycle (Figure S4B). These results indicate that some but not all of the changes in the expression of genes involved in the degradation of specific sources of carbon and nitrogen correlate with the ability of the bacteria to utilize these compounds. Overall, the increased growth capacity of *P. aeruginosa* at 37°C in presence of arginine compared to 22°C, could be related to the ability of this bacterium to adapt to the high amino acid concentrations in the lung during *P. aeruginosa* infection [42]. This metabolic adaptation is achieved independently from previously characterized regulatory mechanisms such as ArgR and Anr.

### Environmental Temperatures Differentially Regulate Pyoverdine and Pyochelin Expression

Iron is a nutrient that plays an essential role as a co-factor in the biochemistry of bacterial cells and regulates the expression of numerous virulence factors [43]–[45]. Its solubility in water is very low, especially at neutral pH and in low oxygen conditions, where Fe^3+^ precipitates [46], [47]. Iron scavenging is essential for bacterial growth, in particular in host settings and in the rhizosphere [48]. *P. aeruginosa* scavenges iron though the secretion of siderophores, such as pyoverdine and pyochelin, that solubilize iron from precipitate or extract it from complexing agents such as lactoferrin [49]. Interestingly, the genes *pvdA* and PA2393 involved in iron acquisition, were found to be down-regulated at 37°C, indicating that *P. aeruginosa* strategy for iron acquisition is associated with temperature and potentially changes upon infection. Most of the genes involved in pyoverdine biosynthesis (*pvdN, pvdF, pvdE, pvdH*) or required for the synthesis of the pyoverdine receptor FpvA (*fpvA*) were up-regulated at 22°C compared to 37°C. The changes of expression of *pvdE* were confirmed by RT-qPCR and the gene was also found to be significantly overexpressed at 22°C compared to 37°C (*p* = 0.0293) (Figure 2). However, the two operons involved in pyochelin expression (*pchDCAB* and *pchEFGHI*) and in the synthesis of the ferripyochelin receptor (*fptA*) were observed to be down-regulated at 22°C compared to 37°C. Similarly, the genes involved in pyoverdine biosynthesis *pvdA* and *pvdH* were also found to be up-regulated in burn wound infections compared to 37°C [16], while genes involved in pyochelin biosynthesis (*pchDCAB*, *pchEFGHI*, and *ftpA*) are down-regulated in burn wound infections compared to 37°C. Pyoverdine biosynthesis genes expression was also observed to be up-regulated at 28°C in *P. aeruginosa* PA14 [13] and in *P. aeruginosa* M18, an isolate from the rhizosphere, compared to 37°C [7], [8]. This result indicates that the regulation of iron acquisition during *P. aeruginosa* transition from the environment to the mammalian host may be associated with temperature changes.

*fur* is a transcription factor and is the master regulator of siderophore biosynthesis and iron acquisition [50], and also controls the expression of pyochelin and pyoverdine biosynthetic and/or receptor genes. In our study, *fur* had similar levels of expression at both temperatures, and no differences were observed in *fur*-controlled genes such as *pvdS, fpvI* and *pchR*. However, the pyoverdine operon regulator *ppyR*, shown to be an activator of pyoverdine biosynthesis genes [51], was up-regulated at 22°C in PAO1 compared to 37°C and might be responsible for the increase expression of the pyoverdine biosynthetic genes and receptor at that temperature.

**Figure 4:**
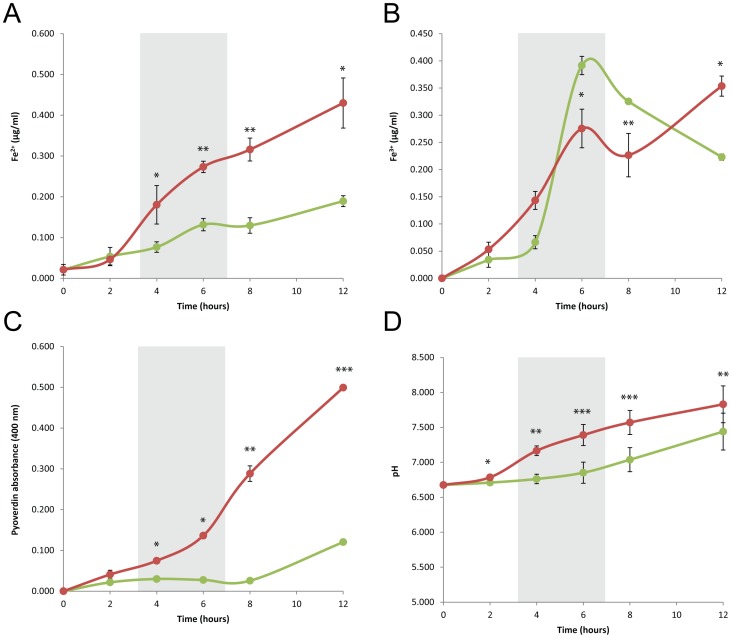
Iron availability, pyoverdin production and pH variation during growth at 22°C and 37°C. Variations of Fe^2+^ (A) and Fe^3+^ (B) levels, pyoverdine production (C) and pH (D) measured during the growth of *P. aeruginosa* PAO1 in Lysogeny broth at 22°C (green) and 37°C (red). Time frames indicated in grey correspond to the times at which samples were taken for transcriptome analysis. Data collected from six independent cultures were analyzed using a paired two-tailed t-test. Data points that were significantly different are denoted with an asterisk.

To better understand the impact of pyoverdine up-regulation and pyochelin down-regulation at 22°C on iron availability, the levels of ferrous and ferric iron were determined in bacterial culture during growth. PAO1 was grown in Lysogeny broth at 22°C and 37°C and samples were taken every two to four hours to quantify the iron present in the culture supernatant. The results obtained show that the release rate of Fe^2+^ and Fe^3+^ are maximal during exponential growth (Figure 4A and B). Whereas Fe^2+^ levels are twice as high at 37°C than at 22°C during exponential growth, Fe^3+^ release in the medium is more than 2.5 fold greater at 22°C compared to 37°C, at time points corresponding to sample collection for gene expression analysis (6 h and 4 h respectively). Fe^3+^ release at 22°C achieves a maximum in mid-exponential phase and is then reduced (Figure 4B). We hypothesize that this result is most likely due to siderophores synthesis shut down to avoid iron overloading and intoxication [52]. Interestingly, the levels of pyoverdine measured in the culture were significantly higher at 37°C than at 22°C (p-values ranging from 0.0170 at 4 h to ≤0.0001 at 12 h) (Figure 4C and Figure S5), and no significant amount of pyoverdine were detected at 22°C until late exponential phase (data not shown). We hypothesize that the activity of the pyoverdine biosynthesis enzymes, such as ferribactin synthase, have a lower enzymatic activity at 22°C compared to 37°C. As a result, the expression of the genes involved in arginine and ornithine degradation for the synthesis of precursors for pyoverdine, together with the genes involved in pyoverdine biosynthesis are up-regulated in an effort to maintain levels of iron appropriate for growth and survival.

Iron availability in solution is tied to the pH of the aqueous environment. Fe^3+^ is insoluble at neutral and high pH, therefore, pH modulation in the growth environment is crucial to maximize iron availability [46], [47]. The variation of pH during growth in Lysogeny broth at 22°C and 37°C was measured. The data obtained indicate that the growth medium becomes more alkaline during bacterial growth (Figure 4D). pH was significantly lower at 22°C than 37°C in mid- and late-exponential phase (*p* values ranging from 0.0136 at 2 h to 0.0006 at 8 h). However the rates of alkalinization of the medium were identical at both temperatures along the growth curve (data not shown).

### Comparison to the Transcriptome of *P. Aeruginosa* at 22°C and 37°C to other Datasets in the Literature

**Figure 5:**
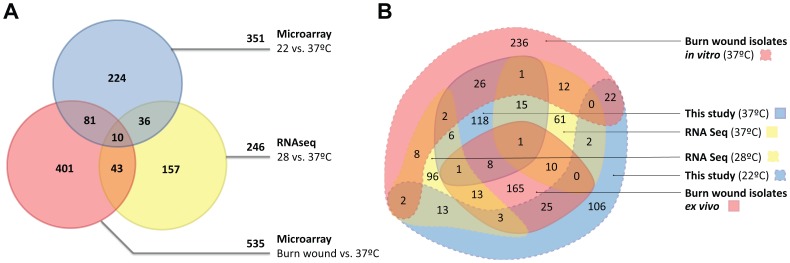
Comparison of three different transcriptomes subjected to environmental temperatures. Venn diagrams comparing datasets proceeding from three different studies: genes observed to be differentially regulated described in this study, genes differentially regulated at 28°C versus 37°C in *P. aeruginosa* PA14 as determined by RNA-seq [13] and genes differentially regulated in *ex vivo* samples of burn wound *P. aeruginosa* infections compared to growth of these isolates at 37°C using Affymetrix microarrays [16]. A. The total number of genes differentially regulated in each dataset is indicated on each arrow. B. The number of genes up-regulated in each condition is indicated in each field. Fold changes in gene expression for the dataset described in this study and the genes present in all datasets are indicated in Table S2 and Table S4, respectively.

*P. aeruginosa* nosocomial infections, such as burn wound and respiratory infections, are principally acquired through contact with contaminated water, medical devices or equipment, or inhalation of aerosols. This process involves the transition of the microorganism from the hospital environment (between 20°C and 25°C) to the skin (28°C to 33°C [53]) or the body (around 37°C). To identify the transcriptional signature associated with the shift from the environment to the host, we compared our results to two different sets of data. The first dataset used for comparison was *ex vivo* gene expression from *P. aeruginosa* burn wound infections isolates [16]. This dataset was used to determine which genes expressed at ambient temperature are required for a successful infection of burn wounds at this temperature. The second set of data was generated using RNA-seq to compare gene expression in *P. aeruginosa* PA14 at 28°C and 37°C [13]. To our surprise, only 10 genes were differentially regulated in all of these datasets (Figure 5A), and only 4 followed the same trend (Table S4). The transcriptional regulator *prtN*, an activator of pyocin biosynthesis [54] associated with niche establishment and protection in mixed environments [55], was found to be up-regulated at 37°C in all datasets. *pvdA* and PA2393, involved in bacterial adaptation and pyoverdin biosynthesis were down-regulated at 37°C in all datasets, indicating that iron acquisition is important in the process of adaptation. PA2411, a probable thioesterase located downstream of *pvdH*, was also found to be down-regulated in all datasets.

To better understand the gene expression overlap in each dataset, a gene onthology (GO) enrichment analysis was performed. The groups compared in this analysis are represented in Figure 5B. Data indicate that genes involved in central energy and carbon metabolism, or in DNA metabolism, transcription and regulation are regulated in all datasets (Table S5). Strikingly, the expression of arginine and glutamine metabolism-associated genes is specific to *P. aeruginosa* grown at 22°C, whereas β-alanine biosynthesis and polyamine catabolism-associated genes are specific to *P. aeruginosa* grown at 37°C (in this study and in [16]). Genes involved in siderophore and pyochelin production are also specific of *P. aeruginosa* both here and in the study by Bielecki *et al.* (Table S5). Interestingly, while the pyochelin synthesis genes *pchABCDEFG* and the pyochelin receptor *ftpA* were found to be up-regulated at 37°C in the current study and in the study by Bielecki *et al.*, the pyoverdine biosynthesis genes *pvdA, pvdF* and *pvdH* and receptor *fpvA* were found to be significantly down-regulated at 37°C in RNA Seq or in isolates proceeding from *ex vivo* burn wounds (Table S5). The genes encoding type I secretion system effectors were found to be exclusively expressed at 37°C here and genes involved in bacterial motility and biofilm formation were only found to be significantly expressed in the burn wound isolates grown at 37°C (Table S5).

Overall, our dataset is more similar to the burn wound infection isolates analysis than to the study using RNA-seq (25.9% and 13.1% of overlap respectively, Figure 5). This result might be due to the fact that our study and the analysis of the burn wound infection isolates were performed using the same technology. The samples were processed using an identical protocol as the one described by Bielecki *et al*. [17], which includes steps of RNA amplification and polyadenylation. This protocol significantly differs from the Affymetrix standard protocol and was used to facilitate the comparison of the data obtained in this study to the data obtained from the burn wound *ex vivo* samples by Bielecki *et al*. [17]. This variation to the protocol might have introduced biases created by differences in amplification and polyadenylation efficiencies. This procedure might be responsible for the higher similarities observed between the dataset in the current study and the burn wound infection isolate analysis [17] compared to the study using RNA-seq [13].

## Conclusion

This work identifies global changes in the expression of gene sets of *P. aeruginosa* induced by a switch in temperature from 22 to 37°C. As hypothesized, the changes in temperature altered the expression of genes found to be involved in bacterial adaptation to the mammalian host. These include quorum sensing-related gene products, such as pyoverdine and pyochelin, and the synthesis of virulence factors, including some proteases and exoenzymes secreted by type I, II, and III secretion systems. Changes in temperature also affected bacterial metabolism, in particular amino acid catabolism and iron acquisition. The changes in genes expression reflect the use of different survival strategies by *P. aeruginosa* in each environment: at lower temperatures, bacterial persistence is favored by a slower and more sustainable metabolism (starch, sucrose and alcohol degradation), whereas at host-specific temperatures, genes involved in virulence and metabolism promoting fast growth (Entner-Doudoroff pathway and TCA cycle) ensure rapid multiplication for host colonization.

The comparisons between the data obtained in this work and in similar studies [13], [16] indicate that there is a high overlap between *P. aeruginosa* gene expression at 22°C and in burn wounds, but that this microorganism has also temperature-specific transcriptional signatures. The comparison of these data might have been greatly influenced by the different analysis methods, platforms, probe specificity or strains used in the different studies. This indicates that choosing the appropriate approach or a combination of them is crucial for the obtaining of biologically representative results. The choice of the method to use is determined by its reliability or the type of data obtained. A broadly used method, such as microarray analysis on commercially available microarrays allows for a good comparison of the data obtained to the published literature. However, there are other layers of regulation that cannot be analyzed using the type of approaches described here. *trans*-acting small regulatory sRNAs, *cis*-antisense transcripts RNAs and riboswitches, play an important role in modulating gene expression [13], and will be considered in future investigations.

Overall, this study provides new insights into how *P. aeruginosa* adapts to temperature changes and identifies some of the factors involved in the adaptation process. This type of study is crucial to help identifying the key factors involved in the discrete steps in infection and to provide new targets for drug development. As systems biology studies continue to refine our knowledge of the metabolic networks of pathogens such as *P. aeruginosa* [56], in the future it should be possible to use these complementary approaches of metabolism and gene expression to precisely identify key factors required for infection.

## Supporting Information

Figure S1 **Growth curves of PAO1 at 22°C and 37°C.** Graphic representation of the absorbance at 600 nm of PAO1 cultures cultivated at 22°C (green) and 37°C (red) in Lysogeny broth under constant sharking. Data represent the average of three independent cultures and a black line shows the absorbance value at which samples were taken for RNA extraction for microarray analysis.
(DOCX)

Figure S2 **Temperature-dependent dysregulation of** ***P. aeruginosa*** **arginine degradation.** Graphic representation of the changes in *P. aeruginosa* PAO1 arginine degradation by arginine succinyltransferase (AST), arginine deiminase (ADI) and arginine decarboxylase (ADC) pathways detected by microarray analysis. Genes up-regulated at 22°C (green) and genes up-regulated at 37°C (red) are italicized next to the reaction catalyzed by their products. Green and red areas represent pathways globally up-regulated at 22°C and 37°C, respectively.
(DOCX)

Figure S3 **Regulation of arginine metabolism in** ***P. aeruginosa*** **.** Graphic representation of the different mechanisms of the regulation of arginine degradation by arginine succinyltransferase (AST), arginine deiminase (ADI) pathways. Genes and operons are represented using a plain arrow and appear in green when they are up-regulated at 22°C. Promoters are indicated with a thin blue arrow in front of the genes. Symbols of (+) and (–) refer to activation or repression of gene expression respectively by the different elements present in this figure.
(DOCX)

Figure S4 **Growth of** ***P. aeruginosa*** **with different sources of carbon and nitrogen at 22°C and 37°C.** Relative growth of *P. aeruginosa* PAO1 at 22°C (green) and 37°C (red) in M9 minimal medium containing 300 mM of arginine (A), succinic acid (B) or glutamic acid (C). Cultures were incubated under constant shaking and the absorbance at 595 nm was determined in volumes of 200 µl. Data were normalized with the absorbance at 595 nm of a culture of PAO1 grown at 22°C and 37°C in Lysogeny broth to correct the differences in growth of *P. aeruginosa* at these temperatures. Data represent the average of three independent cultures.
(DOCX)

Figure S5 **Pyoverdine in culture supernatant after 24 h.** Image of the bacterial culture supernatants of *P. aeruginosa* PAO1 grown at 22°C and 37°C in Lysogeny Broth for 24 h. The assay was setup in triplicate and cultures were centrifuged for 10 min at 5,000 rpm. Supernatants were transferred to a clean tube and the image was taken using a Canon EOS450D of a representative sample.
(DOCX)

Table S1 List of the oligonucleotides used for RT-qPCR.
(XLSX)

Table S2 List of temperature regulated genes on *P. aeruginosa* PAO1 transcriptomes. Table with the complete list of genes up- and down-regulated in the microarrays. Fold changes greater than 1 are indicative or genes up-regulated at 22°C, while fold changes less than 1 are indicative of genes up-regulated at 37°C.
(XLSX)

Table S3 Quorum-sensing and temperature regulated genes. Table with the list of the temperature and quorum-sensing regulated genes identified using Affymetrix microarrays in the study by Wagner *et al*. [33]. Fold changes obtained in both studies are indicated. Fold changes greater than 1 are indicative or genes up-regulated at 22°C, while fold changes less than 1 are indicative of genes up-regulated at 37°C.
(XLSX)

Table S4 **Temperature regulated genes recognized in three different datasets.** Table with the list of the genes commonly dysregulated in the present study, at 28°C versus 37°C in *P. aeruginosa* PA14 as determined by RNA-seq [13] or in *ex vivo* samples of burn wound *P. aeruginosa* infections compared to 37°C using Affymetrix microarrays [16]. Fold changes obtained in this study are indicated. Fold changes greater than 1 are indicative or genes up-regulated at 22°C, while fold changes less than 1 are indicative of genes up-regulated at 37°C.
(XLSX)

Table S5 **GO enrichment analysis of the three different datasets.** GO enrichment analysis output table for the comparison of the genes dysregulated in this study, as determined by RNA-seq [13] or in *ex vivo* samples of burn wound *P. aeruginosa* infections compared to 37°C using Affymetrix microarrays [16]. Data with a *Padj* inferior to 0.05 were considered statistically significant. Abbreviations: Molecular Function (MF), Biological Process (BP), Cellular Component (CC).
(XLSX)
