# Supplementary figures and images for: From the Environment to the Host: Re-Wiring of the Transcriptome of Pseudomonas aeruginosa from 22°C to 37°C
Source: PLoS One. 2014 Feb 24;9(2):e89941. doi: 10.1371/journal.pone.0089941 (PMC3933690; doi:10.1371/journal.pone.0089941)

**Figure S 1. Growth curves of PAO1 at 22°C and 37°C**

**
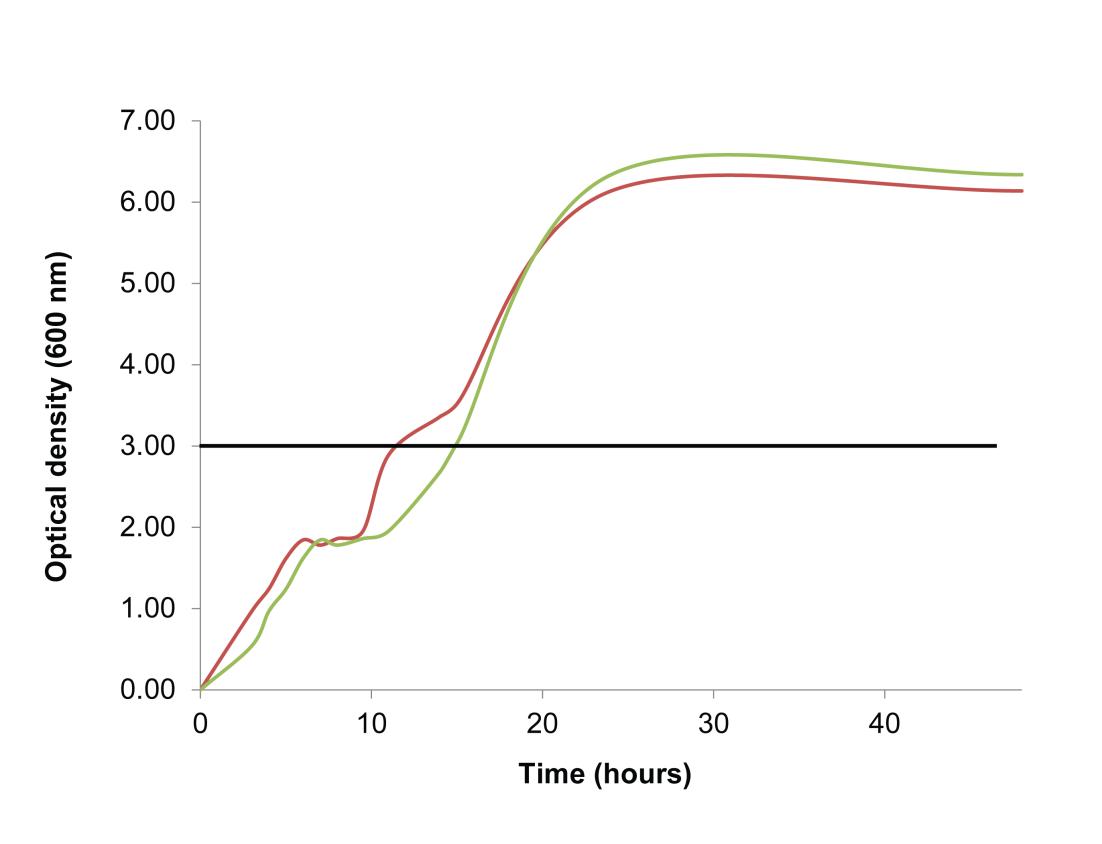
**

Supplement: Figure S1 — Growth curves of PAO1 at 22°C and 37°C. Graphic representation of the absorbance at 600 nm of PAO1 cultures cultivated at 22°C (green) and 37°C (red) in Lysogeny broth under constant sharking. Data represent the average of three independent cultures and a black line shows the absorbance value at which samples were taken for RNA extraction for microarray analysis. (DOCX) [file pone.0089941.s001.docx]

**Figure S 2. Temperature-dependent dysregulation of *P. aeruginosa* arginine degradation.**


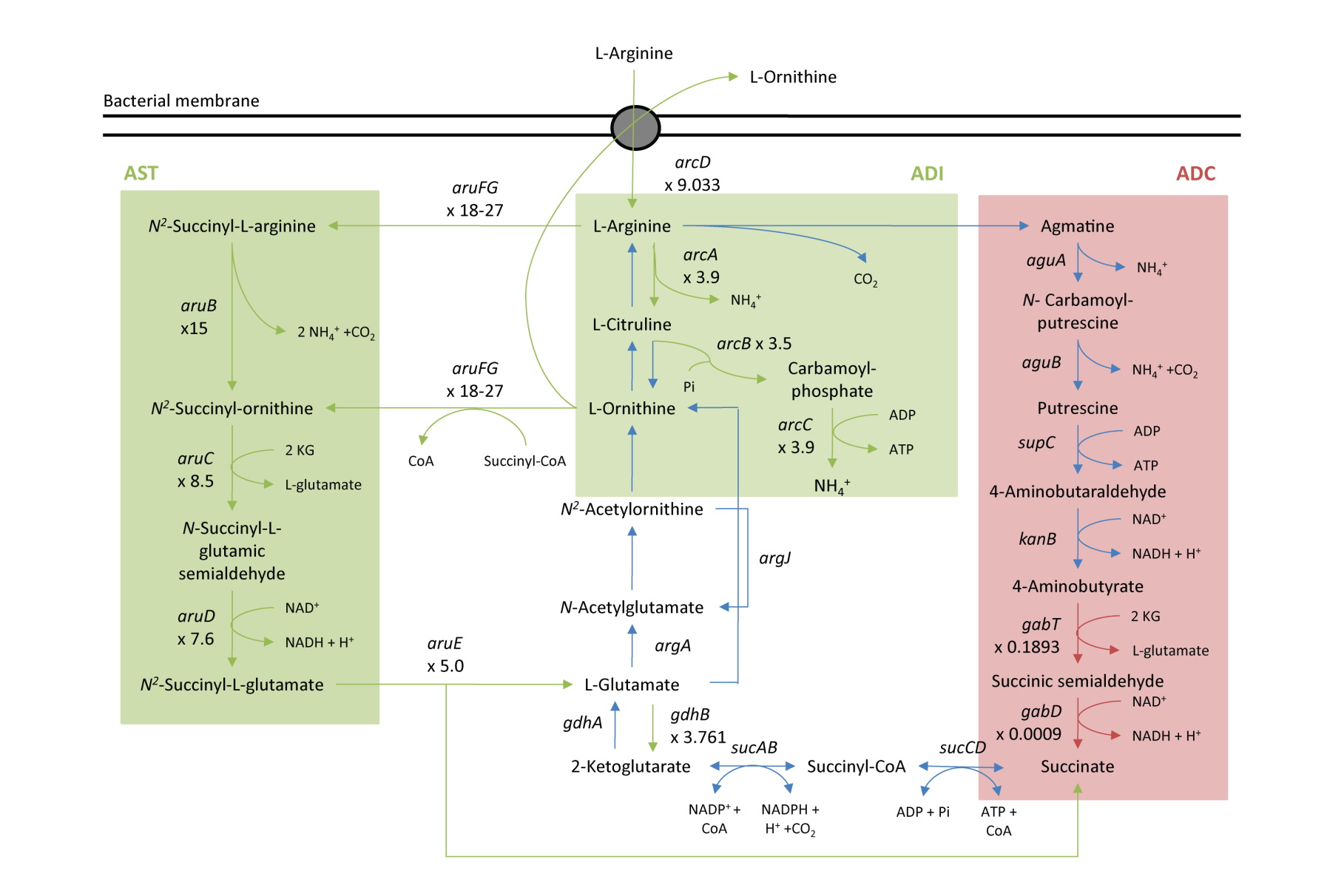

Supplement: Figure S2 — Temperature-dependent dysregulation of P. aeruginosa arginine degradation. Graphic representation of the changes in P. aeruginosa PAO1 arginine degradation by arginine succinyltransferase (AST), arginine deiminase (ADI) and arginine decarboxylase (ADC) pathways detected by microarray analysis. Genes up-regulated at 22°C (green) and genes up-regulated at 37°C (red) are italicized next to the reaction catalyzed by their products. Green and red areas represent pathways globally up-regulated at 22°C and 37°C, respectively. (DOCX) [file pone.0089941.s002.docx]

**Figure S 3. Regulation of arginine metabolism in *P. aeruginosa***


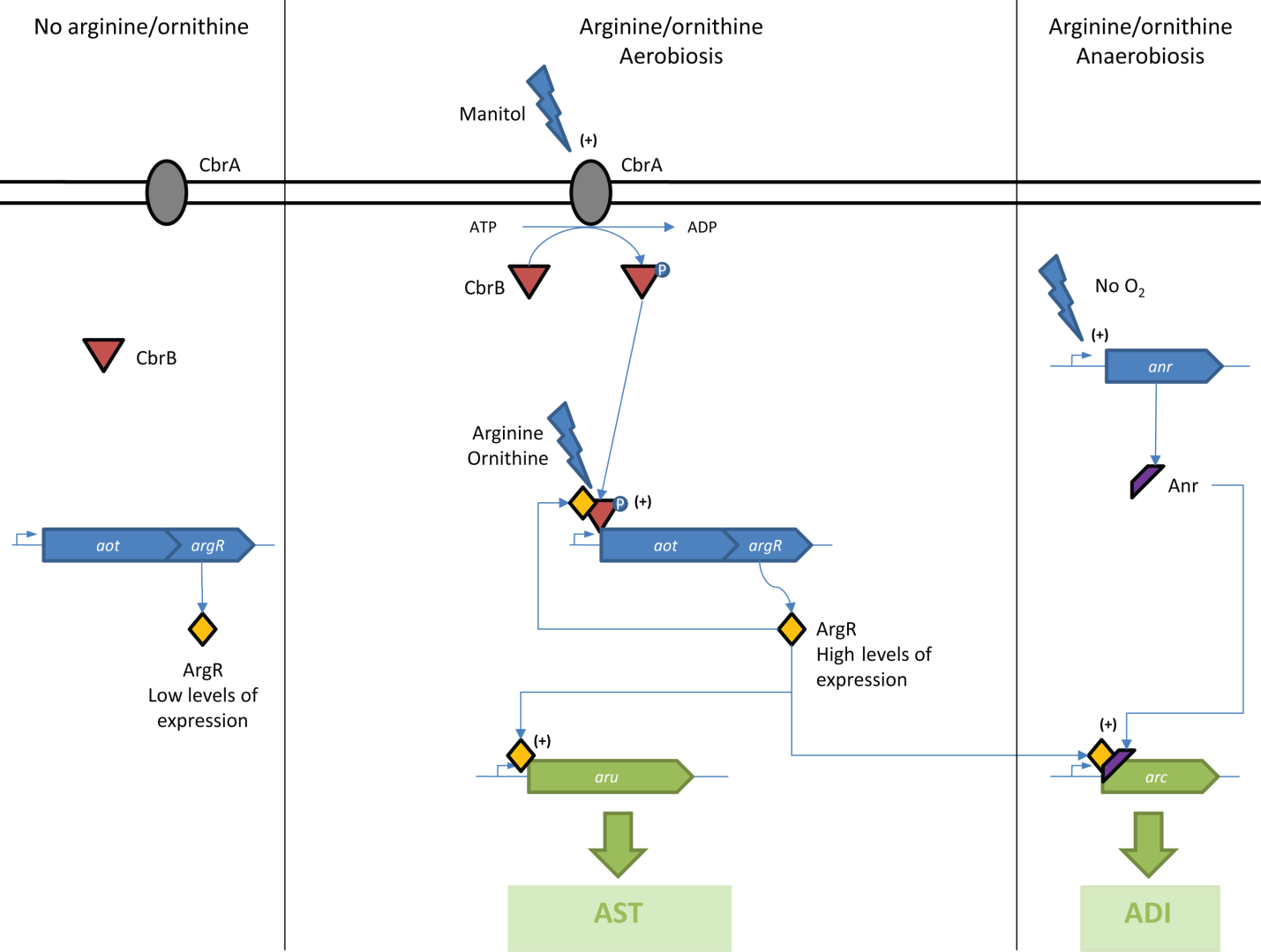

Supplement: Figure S3 — Regulation of arginine metabolism in P. aeruginosa . Graphic representation of the different mechanisms of the regulation of arginine degradation by arginine succinyltransferase (AST), arginine deiminase (ADI) pathways. Genes and operons are represented using a plain arrow and appear in green when they are up-regulated at 22°C. Promoters are indicated with a thin blue arrow in front of the genes. Symbols of (+) and (–) refer to activation or repression of gene expression respectively by the different elements present in this figure. (DOCX) [file pone.0089941.s003.docx]

**Figure S 4. Growth of *P. aeruginosa* with different sources of carbon and nitrogen at 22°C and 37°C**


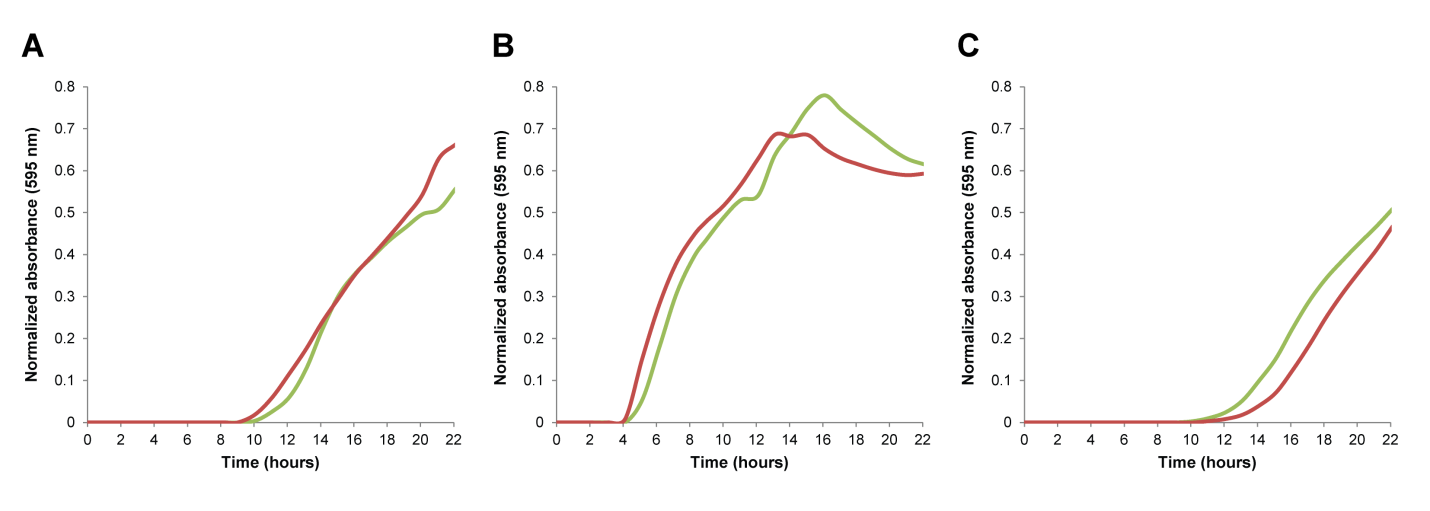

Supplement: Figure S4 — Growth of P. aeruginosa with different sources of carbon and nitrogen at 22°C and 37°C. Relative growth of P. aeruginosa PAO1 at 22°C (green) and 37°C (red) in M9 minimal medium containing 300 mM of arginine (A), succinic acid (B) or glutamic acid (C). Cultures were incubated under constant shaking and the absorbance at 595 nm was determined in volumes of 200 µl. Data were normalized with the absorbance at 595 nm of a culture of PAO1 grown at 22°C and 37°C in Lysogeny broth to correct the differences in growth of P. aeruginosa at these temperatures. Data represent the average of three independent cultures. (DOCX) [file pone.0089941.s004.docx]

**Figure S 5: Pyoverdine in culture supernatant after 24 h**


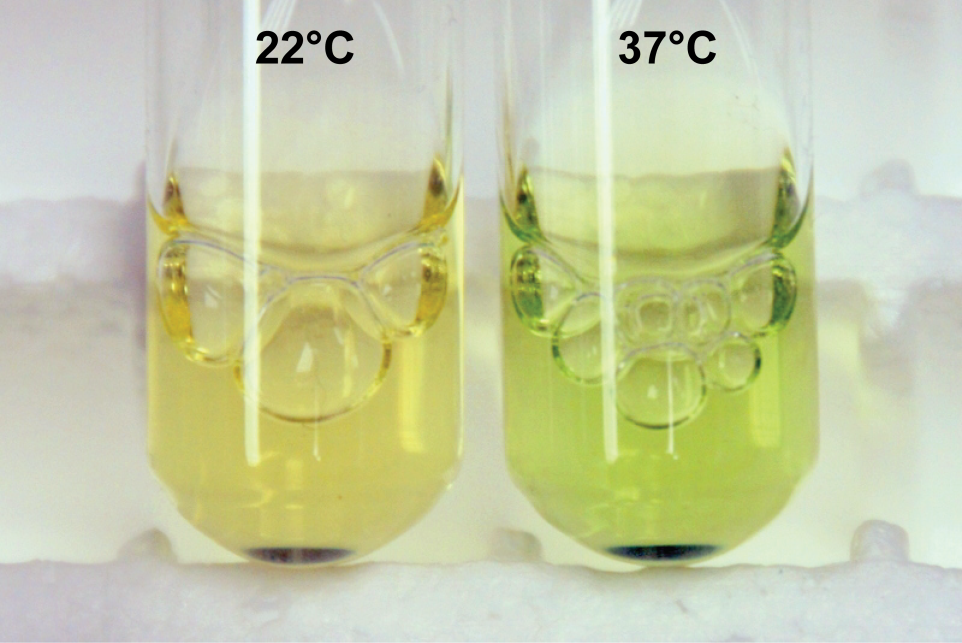

Supplement: Figure S5 — Pyoverdine in culture supernatant after 24 h. Image of the bacterial culture supernatants of P. aeruginosa PAO1 grown at 22°C and 37°C in Lysogeny Broth for 24 h. The assay was setup in triplicate and cultures were centrifuged for 10 min at 5,000 rpm. Supernatants were transferred to a clean tube and the image was taken using a Canon EOS450D of a representative sample. (DOCX) [file pone.0089941.s005.docx]
